# Bone mineral density in university aged Saudi females

**DOI:** 10.12669/pjms.313.7264

**Published:** 2015

**Authors:** Lina Fahmi Hammad

**Affiliations:** 1 Lina Fahmi Hammad, MSc, Ph.D Department of Radiological Science, College of Applied Medical Sciences, King Saud University, Riyadh, Saudi Arabia

**Keywords:** Bone Mineral Density, Osteopenia, Osteoporosis, T-score, DXA, Young Saudi Females

## Abstract

**Objective:**

The aim of the study was to investigate bone mineral density (BMD) in young Saudi females (20-25 years) using Dual-energy x-ray absorptiometry (DXA), as it is a widely utilized modality for the measurement of BMD, used for the diagnosis of osteoporosis and osteopenia.

**Methods:**

BMD measurements were performed in the spine and the neck of the femur in 101 young females.

**Results:**

37% and 34% patients had osteopenia in the spine and the neck of the femur respectively, BMD values = 0.98 and 0.81 gm/cm^2^ respectively). Whereas 5% had osteoporosis in the spine area, BMD values = 0.82 gm/cm^2^). Of the 101 subjects, 53 (52.5%) young females did not suffer from osteopenia in either both site, whereas 23 (22.7%) females had osteopenia in both sites, the remaining 25 subjects (25% of the subject included) had either osteopenia or osteoporosis in one of the sites. A strong association between BMD values with weight was found.

**Conclusions:**

We found that one third of the young Saudi females sampled suffer from osteopenia. Additionally, body weight has a positive relationship with bone mineral density.

## INTRODUCTION

Osteoporosis is a systemic skeletal disorder, characterized by low bone mass, microarchitectual deterioration of bone tissue and an increase in bone fragility and susceptibility to fracture. It is estimated that over 200 million people worldwide suffer from this disease.^1^

Osteoporosis is associated with hip fracture, serious disability and excess mortality.^2^ The world wide annual incidence of hip fracture is approximately 1.7 million, while the annual incidence of distal forearm fractures in males and females was estimated as 1.7 and 7.3 per 1000 persons/years, respectively.^3^ Projections have implied that more than 50% of the world’s osteoporotic hip fractures will occur in Asia by 2050.^1^

WHO reported that osteoporosis was already a global problem and recommended BMD measurements for early detection.^4^ The National Osteoporosis Foundation (NOF) recommended DXA testing of the spine and the hip because these are the most frequent fracture sites.^5^ Several reasons for the popularity of DXA include being able to measure BMD of the posteroanterior spine and hip in a much shorter time than dual-photon absorptiometry, in addition to its capability of measuring BMD of peripheral bones**,** very low radiation doses to the patients, high image resolution, precision, and stable calibration of the instruments.^6^,^7^

There is a need for studies investigating young adults where BMD measurements would reflect peak bone mass and the aim of this study was to evaluate BMD in young university aged Saudi females.

## METHODS

### Study Design

Total 110 young females accepted to participate in the study following the advertisement among the university campus recruiting young females with no previous history of fracture or known bone disease. Each subject was interviewed using a standardized questionnaire, the exclusion criteria included the use of medications affecting calcium metabolism, medical conditions known to affect bone metabolism or with a history of any fracture or major systemic disorder, pregnancy and any terminal illness.

After exclusion criteria were applied, the final population consisted of 101 young (age range 20–24.9 years females). Informed consent was obtained from all subjects. The study was approved by the research ethics committee. Data collected included age, body weight and height and body mass index (BMI) (kg/m^2^) was calculated.

The DXA scans were obtained using Lunar iDXA ™ – GE healthcare, measurements were carried out by 2 experienced technicians in the lumbar spine (L2-L4) and femoral neck area.

### Statistical Analysis

Using SPSS software, version 19.0, (Chicago, IL, USA), One-way Anova test was used to examine differences in BMD between subjects with and without osteoporosis and osteopenia and Pearson Correlation Coefficient was applied to examine the presence of a dependence of BMD and T-score at both the spine and the neck of the femur on age, weight, height and BMI, in subjects with and without osteoporosis and osteopenia.

## RESULTS

Anthropometric data of the subjects studied equaled to (mean age =21.35 ±0.83Yrs, height 1.59 ±0.07m, weight 56.04 ±9.47 kg & BMI 22.27±3.65). BMD values in gm/cm^2^ and T-scores for both sites are presented in Table-I. In the spine, the mean BMD in subjects with a T-score < -2.5 (classified as osteoporotic) was 0.82 gm/cm^2^, compared to 0.98 gm./cm^2^ in subjects with a T-score between -1 to -2.5 (classified as osteopenic) and a BMD value of 1.20 gm./cm^2^ in subjects with a T-score above -1. Similar patterns were found in the neck of the femur, although no subjects had a T-score below -2.5 in that area. Mean BMD in the neck of the femur in subjects with a T-score between -1 to -2.5 (classified as osteopenic) was 0.81 gm/cm^2^, compared to a BMD value of 1.06 gm./cm^2^ in the neck of the femur in subjects with a T-score above -1. Results demonstrated a significant statistical reduction in BMD associated with a T-score equal to -1 and below in both the spine and neck of the femur (P= 0.000).

**Table-I:** BMD and T-score in the spine and the neck of the femur (mean ±SD) in university aged female subjects.

Total Number of subjects (101)			Normal T-score> -1	Osteopenic T-score (-1 to -2.5)	Osteoporotic T-score <-2.5	P-value
BMD	Spine	1.1± 0.2	1.20 ±0.18 (N=59)	0.98 ±0.09 (N=37)	0.82 ±0.19 (N=5)	0.000
	Neck of Femur	0.97 ±0.18	1.06 ±0.15 (N=67)	0.81 ±0.09 (N=34)	- (N=0)	
T-score	Spine	- 0.77 ± 1.06	-0.06 ± 0.7	-1.62 ± 0.37	-2.82 ± 0.19	
	Neck of Femur	-0.65 ± 0.88	-0.15 ± 0.6	-1.63 ± 0.38	-	

Of the 101 female subjects enrolled in the study, 37% and 34% had osteopenia in the spine and the neck of the femur respectively, whereas 5% had osteoporosis in the spine area. Data showed that 58% and 66% of the subjects demonstrating T-score > -1 in the spine and the neck of the femur respectively, which is equivalent to the absence of osteopenia from these sites. Of the 101 subjects, 53 (52.5%) young females did not suffer from osteopenia in both sites, whereas 23 (22.7%) females had osteopenia in both sites. The remaining 25 subjects (25% of the subjects included) had either osteopenia or osteoporosis in one of the sites.

The association between BMD and T-score in the spine and neck of the femur with weight, height and BMI were investigated both in the whole group and in subjects with and without osteopenia in both sites is tabulated in Tables (II, III & IV). Within the whole group, a positive correlation between BMD, T-score obtained from both the spine and the neck of the femur and BMI were found (P <0.001). This finding was a result of the strong correlation of the above parameter (BMD and T-score of the spine and neck of femur) with weight (P <0.001), but not with height (P >0.05). In subjects with no osteopenia in both sites (N=53), such association with BMI was only present in BMD of the neck of the femur (P < 0.05).

**Table-II:** Pearson correlation coefficient of different parameters measured in young females.

Young females (N=101)								
	Age	Weight	Height	BMI	BMD Spine	BMD N.Femur	T score Spine	T score N.Femur
Age								
Weight	0.21*							
Height	-0.03	0.28**						
BMI	0.25*	0.87**	-0.21*					
BMD Spine	-0.01	0.32**	0.01	0.34**				
BMD N.Femur	0.17	0.29**	-0.003	0.31**	0.47**			
T score Spine	0.07	0.39**	0.11	0.36**	0.78**	0.55**		
T score N.Femur	0.1	0.34**	0.17	0.28**	0.63**	0.7**	0.77**	

* Correlation is significant at the 0.05 level.

** Correlation is significant at the 0.01 level.

**Table-III:** Pearson correlation coefficient of different parameters measured in young females without osteoporosis nor osteopenia in both sites.

Young female subjects With T-score> -1 (N=53)								
	Age	Weight	Height	BMI	BMD Spine	BMD N.Femur	T score Spine	T score N.Femur
Age								
Weight	0.13							
Height	-0.16	0.17						
BMI	0.23	0.85**	-0.36**					
BMD Spine	-0.01	0.12	-0.23	0.24				
BMD N.Femur	0.10	0.22	-0.19	0.32*	0.26			
T score Spine	0.15	0.23	-0.06	0.27	0.59**	0.22		
T score N.Femur	-0.002	0.18	0.02	0.18	0.31*	0.39**	0.50**	

** Correlation is significant at the 0.01 level.

* Correlation is significant at the 0.05 level.

**Table-IV:** Pearson correlation coefficient of different parameters measured in young females with osteopenia in both sites.

Young female subjects With osteopenia in both spine and femur (N=23)								
	Age	Weight	Height	BMI	BMD Spine	BMD N.Femur	T score Spine	T score N.Femur
Age								
Weight	0.41							
Height	0.25	0.37						
BMI	0.32	0.92**	-0.03					
BMD Spine	-0.096	0.09	-0.03	0.11				
BMD N.Femur	0.5*	0.09	0.11	0.06	0.22			
T score Spine	0.25	0.17	-0.25	0.28	0.62**	0.31		
T score N.Femur	0.26	0.01	0.41	-0.17	0.32	0.48*	0.32	

** Correlation is significant at the 0.01 level.

* Correlation is significant at the 0.05 level.

The association between BMD and T-score in both sites was investigated both in all female subjects enrolled in the study, as well as in females with and without osteopenia in both sites. A positive correlation in the spine and the neck of the femur in both BMD and T-score were found (P =0.000) within the whole group (N=101) and in the T-score of the spine and the neck of the femur in the non-osteopenic females (P =0.000, N=53).

## DISCUSSION

Bone is a highly specialized support tissue which is characterized by its rigidity and hardness. Bone strength is a composite of bone density and bone quality. Bone density, unlike bone quality, lends itself to easy measurement and is used in the World Health Organization (WHO) operational definition of osteoporosis.^4^ Bone mineral density remains the best available non-invasive assessment of bone strength in routine clinical practice.^5^

Peak bone mass, which occurs during puberty, plays an important role in the determination of osteoporotic fracture risk.^8^ The aim of the study was to provide information about bone density in females at an early stage of adult hood where bone mass gain is at its peak.

Total BMD and T-score values in both the spine and the neck of femur in the present study were lower than values in previous studies, both in Saudi Arabia and the Middle-East area (Table-I). The differences could be attributed to differences in the subjects selection protocol, being in the previous studies excluding subjects with osteopenia or osteoporosis, whereas in the present study being a survey of young female subjects.^9^-^12^

When the data was stratified according to the absence or presence of osteopenia and osteoporosis, no differences in BMD and T-score values were found compared to previous studies. Concentrating on the data obtained in Saudi Arabia, our findings suggest a similarity in BMD values in young subjects without osteopenia in both the central and western region of Saudi Arabia.^11^

The results in Table-I demonstrate the presence of low BMD values in nearly third of young subjects enrolled in the study. These findings are the first within the Saudi population and demonstrate a significant presence of osteopenia (37% and 34% in the spine and the neck of the femur respectively) and osteoporosis (5% incidence in the spine) in young females. Although subjects enrolled in the study stated that they did not suffer from any medical conditions, nor did they take any medication which would affect bone health, the decrease in BMD values reflect a reduction in bone mass that will increase the chance of osteoporotic fracture later in life.

With the diversity of factors affecting bone mass and the presence of more than a third of the study subjects with osteopenia at an early stage of adulthood^13^-^16^, we hope that findings of the study would encourage research on the effects of both genetic and environmental factors on bone density in addition to studying the effect of preventive methods, such as the increase of daily calcium and vitamin D uptake, sun exposure and encouraging exercise at an early stage of life.

The positive correlation found between BMI and BMD in the female group is contributed to the effect of weight and not height on BMD and could be related to the effect of lean and fat body masses on bone density (Table-II).^17^ Results from this study are consistent with previous reports, where increasing weight is known to be associated with higher bone density^9^, which suggests the importance of considering body weight in the evaluation of patients in relation to the diagnosis of osteoporosis^18^, as studies in postmenopausal females recommended that females weighing under 60kg would be a potential candidate for BMD measurement, even in the absence of other risk factors.^19^ Although the present study did not provide data specifically related to fracture risk, reference values should include both healthy young and older subjects in order to determine the peak BMD and, consequently, an accurate estimate of the prevalence of osteopenia and osteoporosis in the population can be obtained.^20^

The positive correlation between BMD values obtained from the spine with T-score in the same region and the similar relationship in the neck of the femur region (Table II, III & IV) suggest that T-score could be used as a tool to educate society members about the importance of bone mineral density measurements. As a result of the numerative range of T-scores (Normal > -1, Osteopenic between -1 to -2.5 and Osteoporotic<-2.5) being easier to be remembered and memorized than the BMD values.

Age-related bone changes demonstrate different patterns; in the spine area, reduction in BMD values were noted (Table III & IV) though not statistically significant, compared to an increase in the BMD at the neck of the femur, which was statistically significant in the subjects with osteopenia only. Differences between our findings and previous studies may result from the wide age range investigated previously, but not applicable in the present study.^11^

## CONCLUSION

The present study shows that third of young Saudi females suffer from osteopenia. BMD values within the central region for young females are comparable to reference data obtained from the western region. Weight has a positive relationship with bone mineral density.
